# The Natural History of Kidney Graft Cortical Microcirculation Determined by Real-Time Contrast-Enhanced Sonography (RT-CES)

**DOI:** 10.1371/journal.pone.0150384

**Published:** 2016-03-07

**Authors:** Carlos Jiménez, María Ovidia López, Amaia Ros, Ana Aguilar, David Menendez, Begoña Rivas, María José Santana, Marco Antonio Vaca, Fernando Escuin, Rosario Madero, Rafael Selgas

**Affiliations:** 1 Department of Nephrology, Research Unit, University Hospital La Paz, IdiPAZ, IRSIN, REDinREN, Madrid, Spain; 2 Department of Nephrology, University Hospital Donostia. Donostia. Spain; 3 Department of Biostatistics, University Hospital La Paz, Madrid, Spain; Hospital Universitario de La Princesa, SPAIN

## Abstract

**Background:**

Kidney transplantation is the therapy of choice for end-stage kidney disease. Graft’s life span is shorter than expected due in part to the delayed diagnosis of various complications, specifically those related to silent progression. It is recognized that serum creatinine levels and proteinuria are poor markers of mild kidney lesions, which results in delayed clinical information. There are many investigation looking for early markers of graft damage. Decreasing kidney graft cortical microcirculation has been related to poor prognosis in kidney transplantation. Cortical capillary blood flow (CCBF) can be measured by real-time contrast-enhanced sonography (RT-CES). Our aim was to describe the natural history of CCBF over time under diverse conditions of kidney transplantation, to explore the influence of donor conditions and recipient events, and to determine the capacity of CCBF for predicting renal function in medium term.

**Patients and Methods:**

RT-CES was performed in 79 consecutive kidney transplant recipients during the first year under regular clinical practice. Cortical capillary blood flow was measured. Clinical variables were analyzed. The influence of CCBF has been determined by univariate and multivariate analysis using mixed regression models based on sequential measurements for each patient over time. We used a first-order autoregression model as the structure of the covariation between measures. The post-hoc comparisons were considered using the Bonferroni correction.

**Results:**

The CCBF values varied significantly over the study periods and were significantly lower at 48 h and day 7. Brain-death donor age and CCBF levels showed an inverse relationship (r: -0.62, p<0.001). Living donors showed higher mean CCBF levels than brain-death donors at each point in the study. These significant differences persisted at month 12 (54.5 ± 28.2 vs 33.7 ± 30 dB/sec, living vs brain-death donor, respectively, p = 0.004) despite similar serum creatinine levels (1.5 ± 0.3 and 1.5 ± 0.5 mg/dL). A sole rejection episode was associated with lower overall CCBF values over the first year. CCBF defined better than level of serum creatinine the graft function status at medium-term.

**Conclusion:**

RT-CES is a non-invasive tool that can quantify and iteratively estimate cortical microcirculation. We have described the natural history of cortical capillary blood flow under regular clinical conditions.

## Introduction

Kidney transplantation is the therapy of choice for end-stage kidney disease when the patient and graft conditions are optimal. Despite significant therapeutic and immunological advances, the graft’s lifespan is shorter than expected due in part to the delayed diagnosis of various complications, specifically those related to silent progression [1–3]. Serum creatinine levels and proteinuria are the most frequently used markers; however, both share the same limitations: they are markers of established advanced or nonreversible lesions. It is recognized that serum creatinine levels and proteinuria are poor markers of mild kidney lesions, which results in delayed clinical information. Minor graft lesions might not induce changes in any of these markers due to adaptive functional changes. In contrast, iterative graft biopsies are much more informative but are limited by their invasive character [2,4,5]. New intermediate markers for these conditions have been proposed by transplantation groups who believe that earlier lesion markers will enable more effective medical management and better long-term transplantation outcomes.

Renal cortical microcirculation is recognized as an important part and target in the response of a grafted kidney and especially affects chronic damage [2]. In other conditions, cortical microcirculation damage (confirmed by renal immunohistochemical data) has been related to poor prognosis in a number of diseases [6,7]. Reduced microvascular flow (represented mainly by the loss of the peritubular capillary network)consists of the perfusion of various parts of the nephrona and causes dysfunction and loss of the organ. The first step of chronic allograft humoral rejection is mediated by peritubular capillary inflammation [8,9].

Our hypothesis is that numerous graft lesions start by affecting the vascular network [8,10,11](simultaneously affecting other nephron structures or not) but with effects that are potentially more demonstrative by means of direct measurements. We also hypothesize that mild change in normal renal function markers might not predict the long-term effects of some lesions, whereas cortical microcirculation parameters could be more sensitive in expressing the lack of healthy tissue reserve. If this is the case then changes in graft microcirculation could predict graft status more accurately than serum creatinine levels and proteinuria in the long term.

Cortical capillary blood flow (CCBF) can be measured by real-time contrast-enhanced sonography (RT-CES), a technique that has recently been made available. This technique provides an analysis of vascular refilling in any region of interest (e.g., the renal cortex). A perfusion study can be performed by continuously injecting microbubbles, which are then destroyed by an ultrasound pulse with a high mechanical index. Vascular refilling can also be quantified by performing time-intensity curves using a software tool. The refilling of an area is therefore a marker of tissue perfusion [12,13]. Its accuracy has been demonstrated in human and animal models, [14–17]and a number of studies have been published on this subjectin the field of renal transplantation [18–23]. The image quality, the repeated measurements and the lack of nephrotoxicity of this procedure put it in competition with computed tomography and nuclear magnetic resonance. Doppler ultrasonography is appropriate for measuring arterial blood flow in large arteries but is not so effective for the renal cortex.

The primary aim of our study was to describe the natural history of cortical capillary blood flow to explore the various patterns of this parameter over time under diverse conditions of kidney transplantation. A secondary study objective was to explore the influence of donor conditions and recipient events (acute rejection, acute tubular necrosis, calcineurin inhibitor toxicity) on CCBF. The third objective was to determine the capacity of CCBF for improving the predictive value of current plasma creatinine levels in the medium term.

## Patients and Methods

### Study Population

We evaluated all patients who underwent kidney transplantation in our unit from 2009 to 2010 by RT-CES, with no other inclusion/exclusion criteria. Table 1 lists the main characteristics of this series. All available patients at each moment were studied after 48 h, 5–7 days, and 1, 3 and 12 months after transplantation.

**Table 1:** Demographic and Clinical Characteristics of the Patients and Allografts (n = 79).

**Donor-related factors**	
Age (years)	49.5±13.2
Deceased / living donor— no. (%)	68 (86) / 11 (14)
Hypertension (%)	16 (21.3)
**Recipient-related factors**	
Age at transplantation (years)	52.1±14.4
Gender-male—no. (%)	51 (63.8)
Previous renal transplantations—no. (%)	15 (18.9)
Prior Time on dialysis (months)	39.5±26.8
High immunological risk—no. (%)	12 (15.2)
HLA mismatches	4±1.4
Cold-ischemia time—hr (deceased donor)	14.1±4.5
Delayed graft function—no (%)	10 (12.5)
Acute rejection–# (%)	8 (10.1)
Anticalcineurinics drugs—no (%)	
Early tacrolimus	36 (45.5)
Delayed tacrolimus	43 (54.4)

The study was approved by our institution’s ethics committee (Comité Ético de Investigación Clínica del Hospital Universitario La Paz). Patient records/information was anonymized and de-identified prior to analysis.

### Immunosuppression

Immunosuppression is a key study variable, considering its ability to induce changes in the renal vascular network by calcineurin inhibitors [20,21]We therefore established this treatment as a major condition in our examination. Thirty-six patients were treated with tacrolimus from the first day, as well as steroids, mycophenolic acid or mycophenolate mofetil (MMF). Basiliximab or thymoglobulin was administered as induction therapy, depending on the immunological risk. Due to their higher risk of ischemia/reperfusion injury, 39 other patients were administered once-daily formulation tacrolimus when their renal function clearly started to improve or otherwise on day 7. All of these patients were also treated with thymoglobulin, steroids and MMF from day 1.

The maintenance immunosuppression therapy also included steroids and MMF, adjusted according to the patient and graft outcomes.

### Clinical Variables

The study variables were obtained from our renal transplant data base and included the following:

- Donor: origin (living donor, brain-death or circulatory-death donor), donor cause of death (trauma vs. medical), age, sex and history of hypertension and diabetes.
- Recipient: age, sex, type of and time on dialysis, kidney disease diagnosis, other previous kidney grafts, warm and cold ischemia times, immunosuppressive drugs (see prior section), delayed graft function (defined by the need for dialysis), acute rejection episodes(demonstrated by graft biopsy), serum creatinine follow-up, glomerular filtration rate(determined by Modification of Diet in Renal Disease [MDRD]), proteinuria and trough serum tacrolimus levels coinciding with the sonography, number of HLA-A, B and DR loci mismatches and percentage of panel reactive antibodies.

### Sonography (RT-CES)

**RT-CES** consists of B-mode sonography with Doppler and contrast administration. Sonography was performed with an Acuson Sequoia ultrasound system and a 4C1 convex transducer (Siemens Medical Solutions, Madrid, Spain), at a frequency of 1.5 MHz in transmission and reception. Initial kidney visualization was performed in a sagittal imaging plane at 3.5 MHz with a mechanical index (MI) of 1.

Initially, conventional graft B-mode sonography and Doppler scan were performed. Measurements of the graft (length, width and renal parenchyma width) were performed. The resistance index was obtained from the area of the proximal segmental arteries (the first vessels branching off of the main renal artery).

Contrast-enhanced sonography was then performed with Cadence contrast pulse sequencing (CPS) at a low MI (0.2–0.5). Microbubble destruction was performed in the Cadence CPS imaging mode by applying an MI of 1.9 with a continuous frame rate of 14 Hz. Information about the Cadence output running in the CPS imaging mode at an MI of 1.9 is as follows: peak rarefactional pressure, 2.1 MPa; distance from the transducer output face to the point of maximum pulse pressure squared integral, 87 to 38 mm; and spatial-peak temporal-average intensity, 607 mW/cm^2^. Scanner calibration was performed according to manufacturer specifications following the International Electrotechnical Commission’s recommendations. The gain settings, depth and focus were optimized and maintained constant throughout each study.

The transducer was held in a fixed position to scan the graft in a longitudinal cut. When major kidney structures were barely visible, a contrast agent was applied. Each patient was administered a contrast agent (sulfur hexafluoride, SonoVue^®^; Bracco SpA, Milan, Italy) through the peripheral vein in a continuous perfusion (Vueject BR-INF 100; Bracco SpA) with constant agitation. The initial infusion (4 mL of SonoVue in 16 mL of saline) was performed at 4 mL/min. Optimal kidney graft contrast visualization was usually achieved at 30–60 seconds.

**Fig 1:**
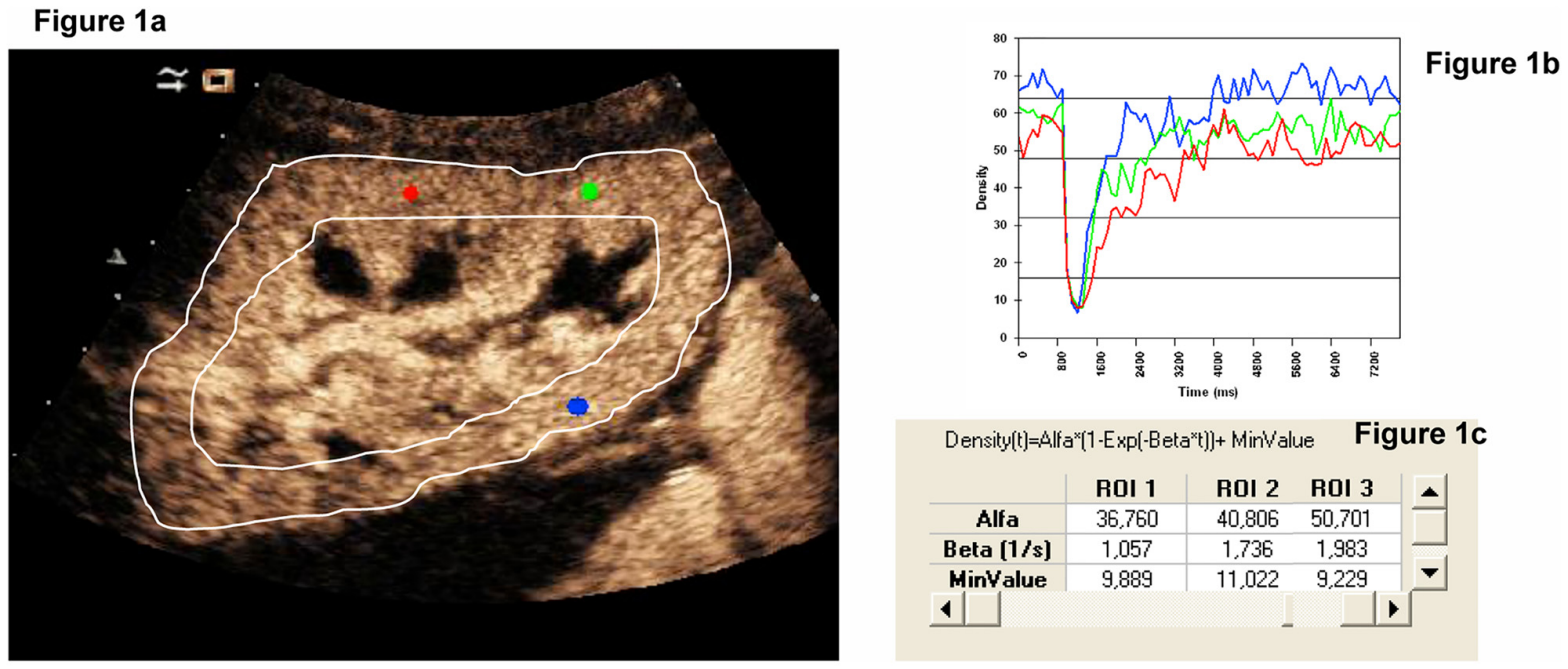
This figure shows renal graft perfusion by contrast media in a longitudinal view. Red, green and blue circles are regions of interest selected in the proximal and distal renal cortex. The white line identifies and isolates the renal cortex. Once uniform graft perfusion was achieved, microbubbles were destroyed with a high mechanic ultrasound pulse. The replenishment kinetics was recorded stored in order to perform time-intensity curves using a software tool (Fig 1b). Cortical capillary blood flow for each region of interest could be estimated (Fig 1c).

After verifying uniform perfusion in the kidney, a 1-second microbubble destruction was performed at a high MI (MI:1.9), which provided time-intensity curves using the software tool (Fig 1). The replenishment kinetics was digitally recorded for 20 seconds in order to create time-intensity curves. After this first examination, 2–5 consecutive procedures were performed in the same scan plane, resulting in 2–5 replenishment sequences for each patient.

Quantitative analysis of renalcortical perfusion was performed using a software tool (CUSQ 1.4^®^; Siemens Medical Solutions). Three circular regions of interest (ROI) were selected in the subcapsular renal cortex, 2in the renal cortexmore proximal to the transducer and 1in the contralateral renal cortex (Fig 1). Interlobular and arcuate arteries were carefully excluded from the region of interest. Cortical capillary blood flow (CCBF) is expressed as decibels/second. Contrast intensity was measured after calculating flash and spline curves of contrast intensity versus time, which were determined according to the following exponential function:y = A x (1- e–ßt) [13]. We calculated the product A x ß was calculated, which defines capillary blood flow in the renal cortex (A represents the plateau of the video-intensity signal and ß the slope of the maximum video-intensity signal). The CCBF of each patient was calculated as the mean of the 3 selected ROIs.

Ultrasonography was performed by at least 2 technicians using the same protocol. The quantitative analysis of CCBF was also performed by 2researchers.

### Inter-Observer Variability

In a previous study [24], we calculated the interobserver reproducibility of the quantitative analysis by means of 2 blinded and independent observers performing the CCBF quantification on recorded images. Previously, both investigators established the 3ROIsto be selected, 2in the proximal cortex and the other in the deep renal cortexes.

The microvascular quantification calculated by the 2 observers showed no significant differences. In the reliability analysis, the mean intraclass correlation was 0.81 (F, 9.88; p = 0.0001) for a 95%confidence interval. So, in this actual study, interobserved reproducibility was not performed because methodology was similar to the 2007 study.

### Statistical Analysis

Data are shown as mean ± SD and medians and quartiles are employed when there is a non-normal distribution. Qualitative variables are expressed as percentages. The influence of CCBF has been determined by univariate and multivariate analysis using mixed regression models based on sequential measurements for each patient over time. We used a first-order autoregression model as the structure of the covariation between measures. The *post-hoc* comparisons were considered using the Bonferroni correction.

Serum creatinine was selected as the prognosis marker for graft outcome following the recommendations of several authors [25–27]. The CCBF values with the corresponding serum creatinine values measured simultaneously were compared in terms of prognosis.

## Result

During this period, 89 kidney transplantations were performed in our institution. Ten patients were excluded from the longitudinal study due to premature graft loss (during the first 15 days):two grafts from circulatory-death donors never functioned. Five grafts (one of then from circulatory-death donor) suffered graft thrombosis in 24 hours. One recipient suffered immediate hemorrhagic complication and required transplantectomy before 24 hours. A recipient died because of myocardial infarct at day ten with very good graft function. One recipient suffered delayed graft function and humoral acute rejection and never functioned (a transplantectomy was performed).

As a result, 79 grafts (4 circulatory-death, 64 brain-death and 11 living donors) were finally studied since the aim of our study was to explore graft CCBF over time more than observe the immediate values. Patient characteristics are shown in Table 1.

### The Natural History of CCBF

**Fig 2:**
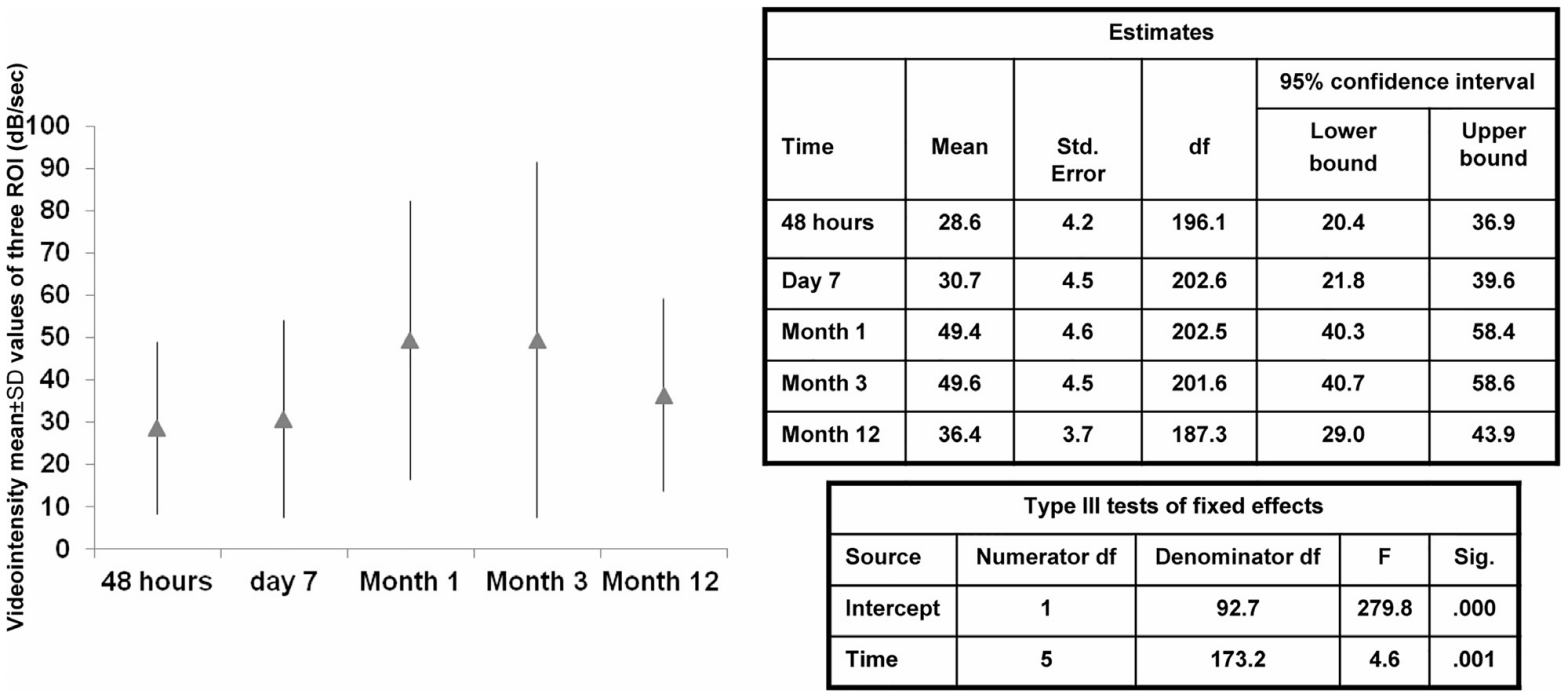
Estimated CCBF mean values of the 79 patients studied at various times after transplantation. It is notable that the CCBF starts at lower levels than those that will be reached at months 1 and 3 (this peak varies among patients). It is also remarkable that, after 12 months, CCBF values decrease significantly, remaining at an intermediate level between the initial and maximum values.

**Fig 3:**
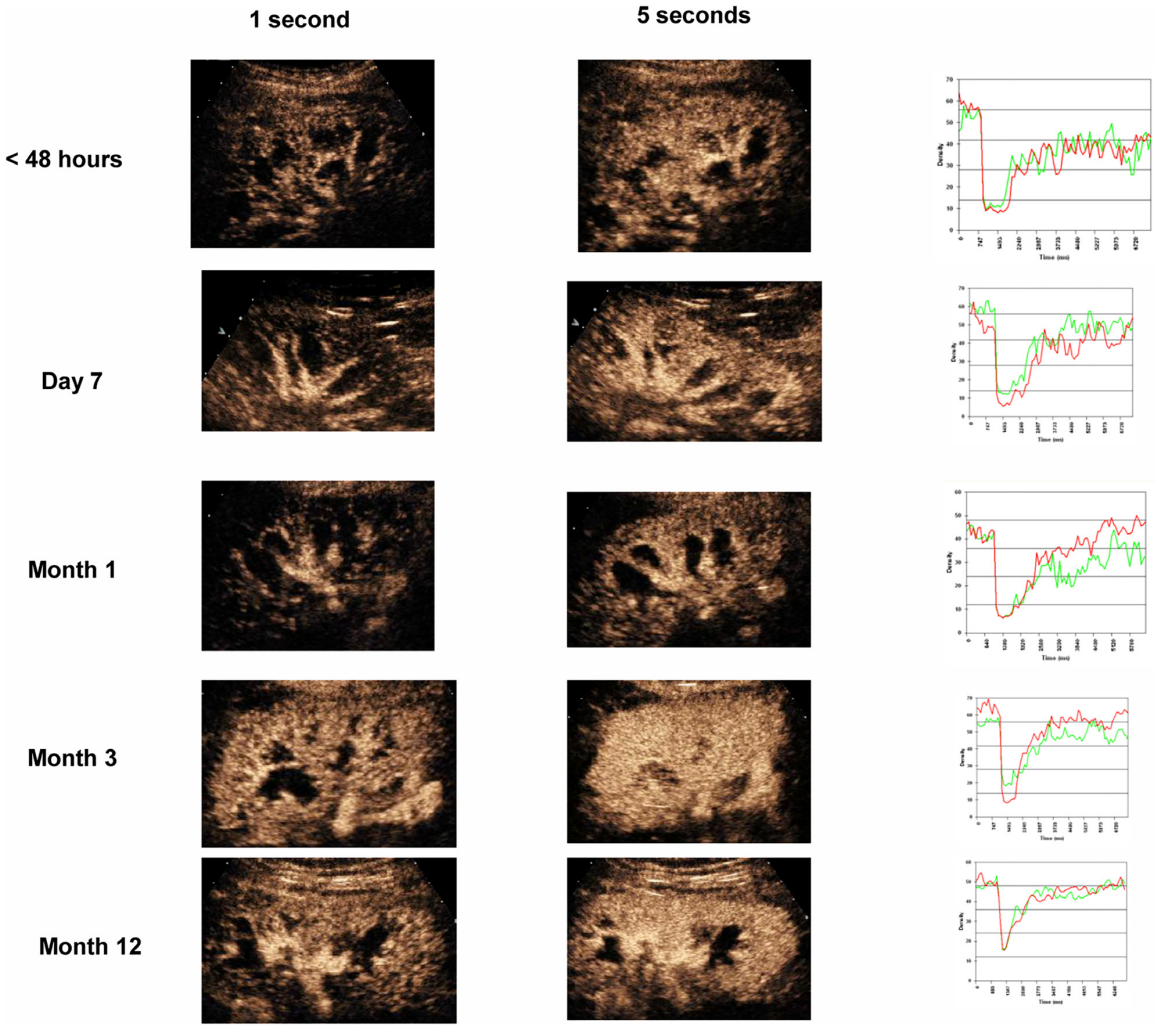
Actual example of the studies performed in a brain-death donor recipient over time. RT-CES images obtained at 1 (a) and 5 (b) seconds from refilling after bubble destruction at various times 1 year after transplantation. On the corresponding right side, flow-volume curves obtained at 2regions of interest (red and green) are shown (c). Initial image (1a and 1b) demonstrates obvious renal flow in the large vessels (a and b) and renal cortex (b). At days 7 and 30, these images are very similar, reflecting the same structures at each moment. However, images at month 3 show early cortical flow (4a) not present in prior examinations(1–3 a), demonstrating faster cortical refilling. Very similar features are found at month 12 (5). Images 1-5c show flow-volume curves that reflect the number of cortical capillaries in the refilling phase. There is a notable correspondence between the 2 measurements along all studies (red and green) and the differences in the area under the curve (AUC) between 1c and 2-5c.

We attempted to define the natural history of CCBF using the results of examinations performed during the first week and at months 1 to 12 after transplantation; these values are shown in Table 2. The CCBF values varied significantly over the study periods and were significantly lower at 48 h and day 7 than at months 1 and 3. The mean CCBF value at month 12 was 36.7± 22.7 dB/s, with minimum and maximum values on day 7 and at months 1and3, respectively. The CCBF value followed a mostly ascending statistically significant slope, with a peak at month 1–3, followed by a partial descent (relative to baseline) until month 12(Fig 2).Table 2 lists the CCBF values expressed as dB/sec at various times since transplantation. As can be seen by the results, there is a wide interpatient variability. Fig 3 shows an actual example of the studies performed on a brain-death donor recipient over time. The results of both imaging and area under the curve studies (2flow-volume curves were obtained at 2regions, red and green) coincide and reflect the different vascular perfusions exhibited by the same graft in its various regions over time. More details can be observed in the videos included in S1 video.

**Table 2:** Estimated cortical capillary blood flow (CCBF) values expressed as db/sec at different times after transplantation.

	48 hours	Day 7	Month 1	Month 3	Month 12
Patients included	45	36	35	35	57
Mean	28.6	30.7	49.4	49.6	36,4
Median	26.3	23.8	42.4	36.4	31.5
SD	20.3	23.3	33	42	22.7
Minimun value	3.3	3.6	10	8	5.9
Maximun value	101.8	96.2	142	229.1	103.4
25 Percentile	12.3	14.5	23.1	22	22.9
75 Percentile	40.7	36	64	67	42.9

### Influence of Donor and Recipient Conditions on CCBF

The natural history of CCBF will depend on donor conditions and the subsequent influences on the recipient.

### CCBF and Donor Characteristics

**Fig 4:**
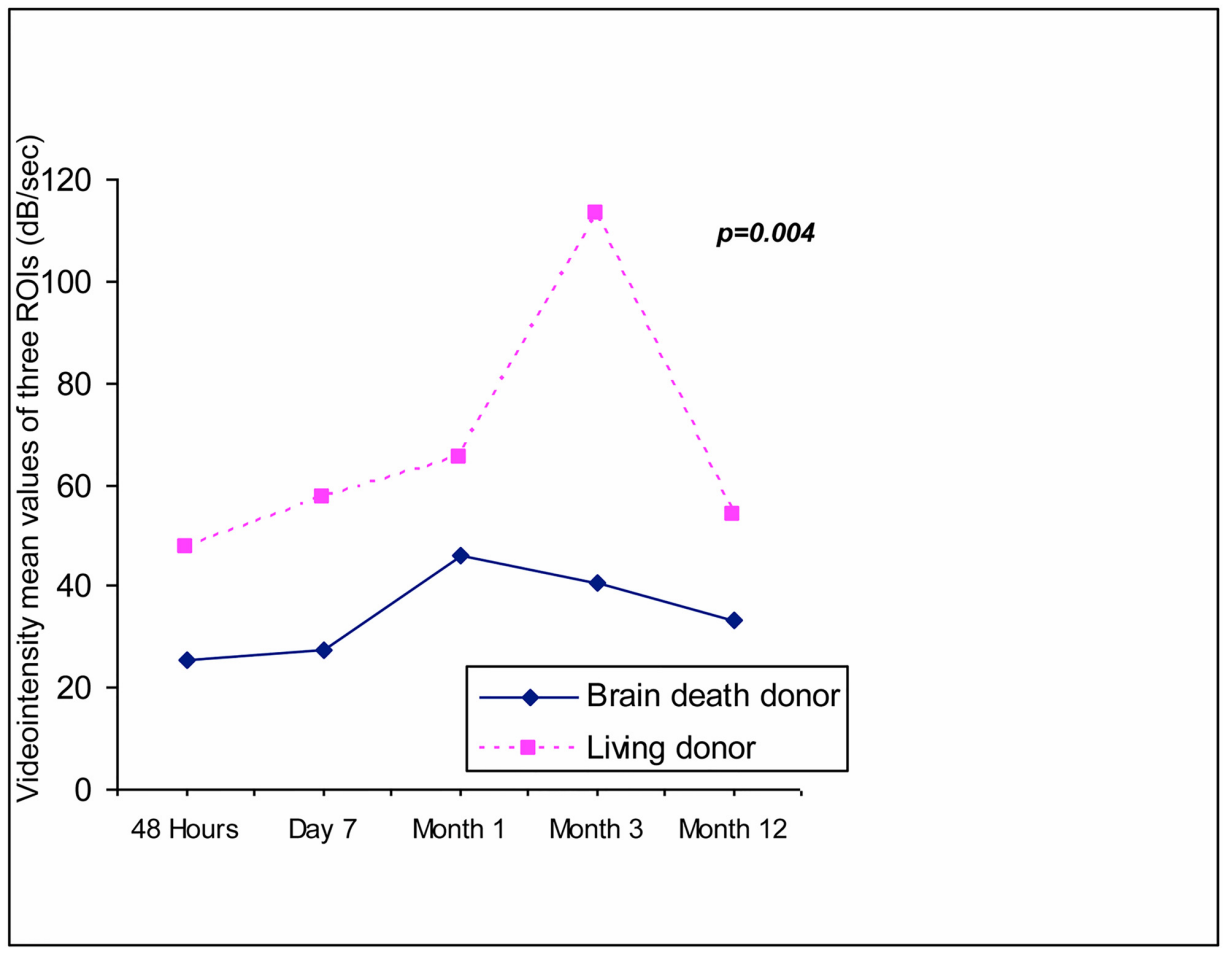
Using mixed model analysis, the CCBF values during follow-up were significantly higher among grafts from living donors when compared with brain-death donors.

**Fig 5:**
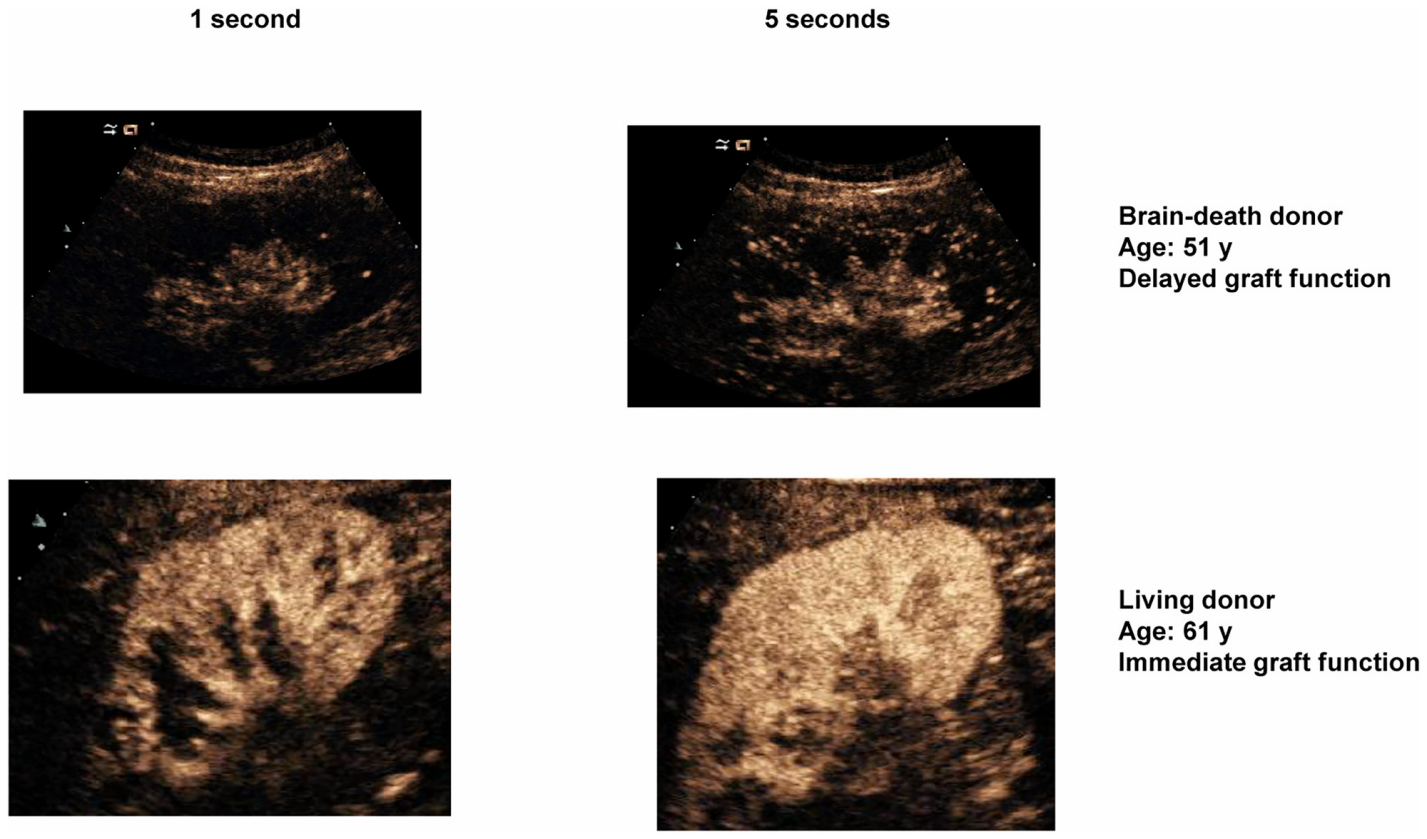
This figure shows the examination performed in the first 48 h of grafting of two representative patients. The first was a brain-death donor with acute tubular necrosis and the second was a living donor. We observed the obvious differences between patients: Graft microcirculation from living donor was quicker and more intensive that graft microcirculation from brain-death donor.

We observed that grafts from living donors showed significantly higher mean CCBF levels than grafts from brain-death donors at each point in the study (Figs 4 and 5). From a predictive point of view, these significant differences persisted at month 12 (54.5 ± 28.2 vs 33.7 ± 30 dB/sec, living vs brain-death donor, respectively, p = 0.004) despite similar serum creatinine levels (1.5 ± 0.3 and1.5 ± 0.5 mg/dL for living donor and brain-death donor, respectively, ns), demonstrating the different expression of the 2markers, depending on donor conditions.

In contrast to brain-death donors, it is remarkable that circulatory-death donor grafts showed an initial CCBF(2–7 days) value similar to that of living donors and significantly higher than that of brain-death donors. Two of these grafts never functioned and were excluded from the analysis. The other 4 showed delayed graft function (DGF) and were followed by renal biopsy per protocol. CCBF values of these 4graftsshowsimilar values to that of living donors at day 7 and month 12. Median CCBF values were 44.5 at day 2, 45.6 at day7 and 49.8 dB/sec at month 12.

Acute tubular necrosis, present in brain-death and circulatory-death donors, was not differentiated except for the different CCBF levels, representing an added value for the examination of these uncertain situations that usually require renal biopsy.

**Fig 6:**
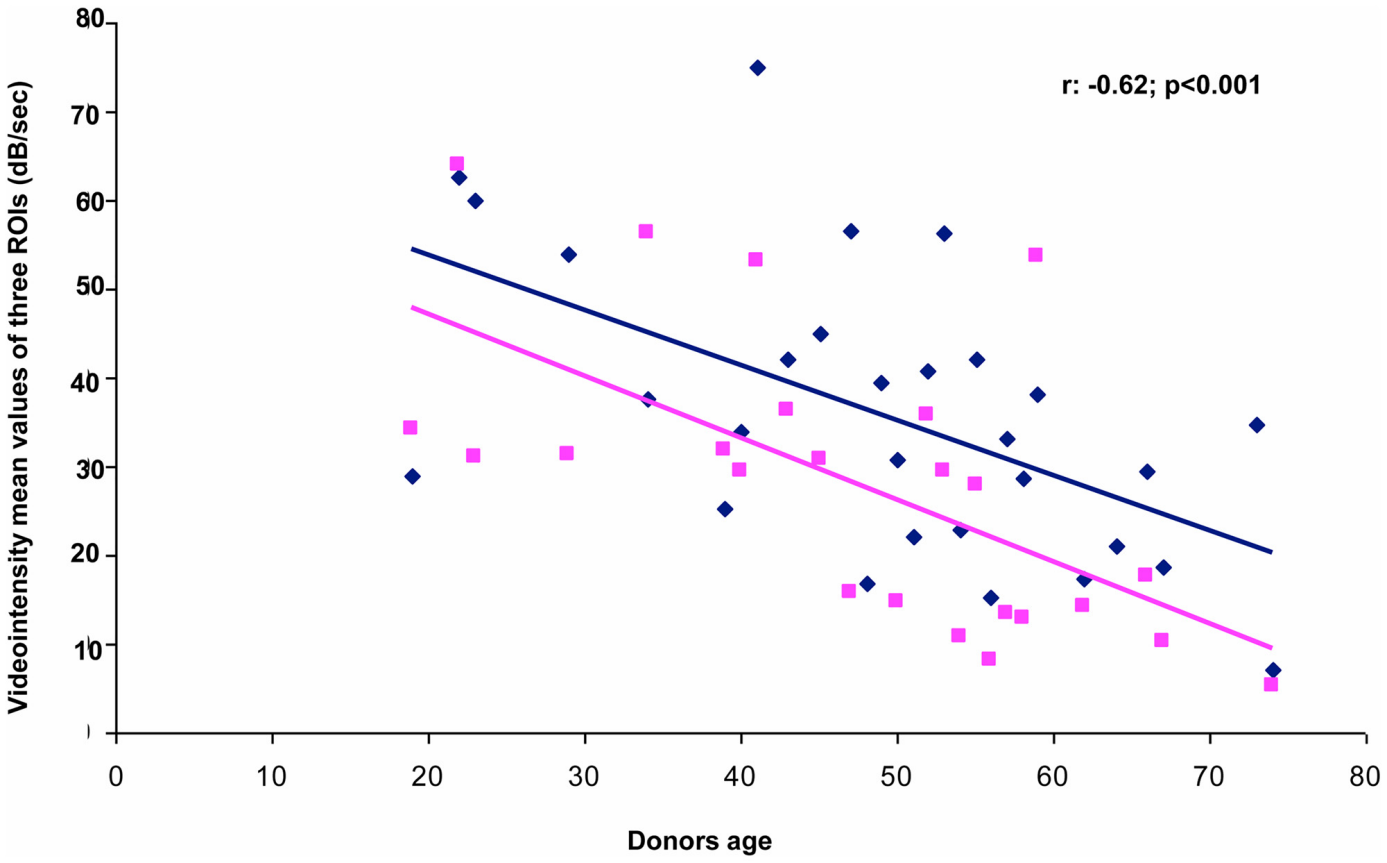
Significant relationship between donor age and CCBF level at 48 h(square points), and the average CCBF value over the whole follow-up (diamond points; r, -0.62; p<0.001).

We observed that brain-death donor age and CCBF levels showed an inverse significant relationship (r: -0.62, p<0.001) (Fig 6). This relationship was present from the examination in the first 48h after graft implantation to when further examinations were conducted.

Brain-death donors younger than 40 years showed CCBF values that were no different from that of living donors at almost all study stages, in contrast to the lower values showed by donors over 59 years of age.

Other donor conditions were also analyzed (cause of death, prior arterial hypertension, diabetes) although there was no significant relationship between any of these conditions and CCBF.

### Recipient Conditions

Neither the type of dialysis nor the cold ischemia time showed any correlation to CCBF. The presence of DGF did not significantly affect the CCBF curve morphology over the first year, although these values were slightly higher at months 3 and 12 in the non-DGF group (mixed models).

Immunosuppression induction: Given that the use of calcineurin inhibitors can affect CCBF levels, we compared CCBF values under early and delayed use (these recipients were treated with Thymoglobulin). Although it is our practice to prescribe thymoglobulin for higher risk patients with DGF, the data demonstrated a higher CCBF value over the first month relative to those taking tacrolimus since the first day (considering these recipients also had a lower DGF risk). In contrast, patients who started tacrolimus at day 1 demonstrated a transient decrease in CCBF levels during the first week. This observation suggests the possibility of vessel constriction induced by tacrolimus. No differences in terms of CCBF were observed between these groups at month 12. We observed no correlation between CCBF levels and simultaneous tacrolimus trough plasma levels at any time.

**Fig 7:**
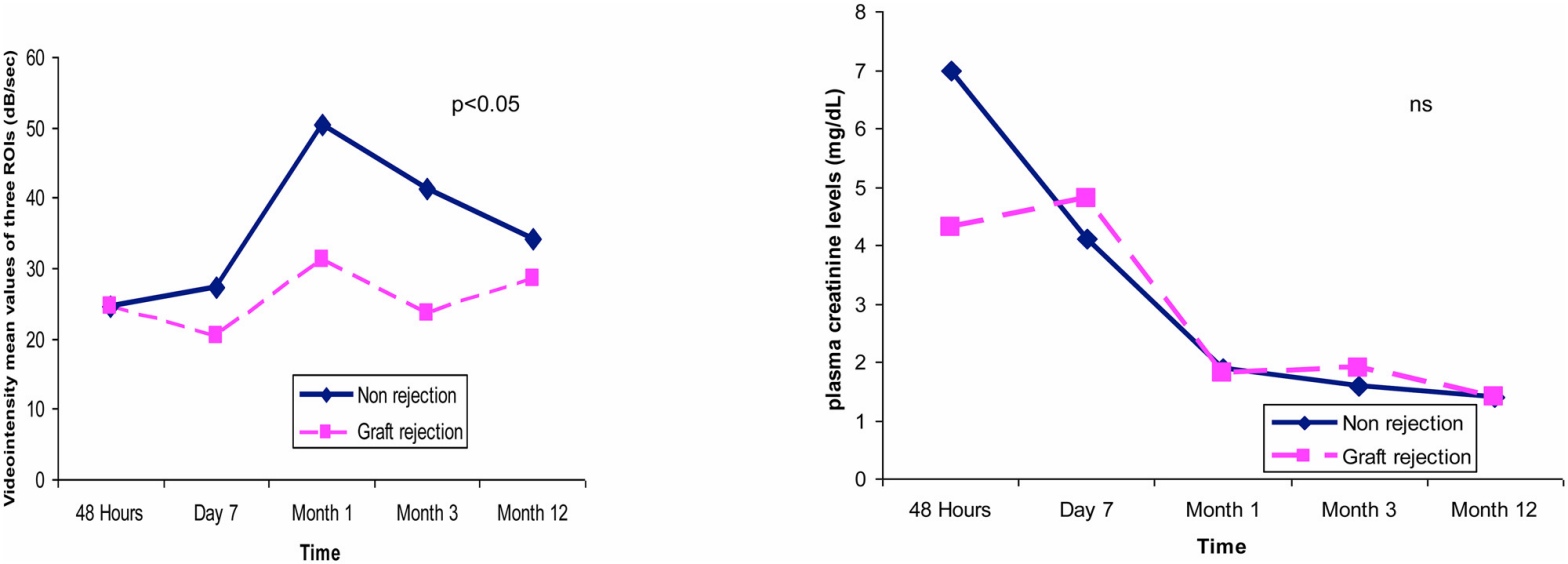
In the left panel, the CCBF values for patients with (green) and with no acute rejection demonstrate significant differences by mixed model analysis over time. In contrast, plasma creatinine levels (right panel) exhibited no differences for the same patients.

Eight cases had an acute rejection episode demonstrated by a renal biopsy. Coinciding with these episodes, CCBF showed non significant lower values relative to patients not experiencing graft rejection. Furthermore, the presence of a rejection episode was significantly associated with lower overall values over the first year (Fig 7, left). There is a notable discrepancy between CCBF and serum creatinine values at month 12, given that the latter value showed similar levels for both groups(rejection vs. no rejection) (Fig 7, right).

To compile the data obtained from donor/recipient conditions and their relationship to the CCBF value at various times, we performed a multivariate analysis. There was an independent significant relationship between CCBF and donor type (living vs. brain-death), donor age and serum creatinine level. The presence of acute rejection was almost significant, but the other variables did not remain in the model. Table 3 displays the details of this relationship.

**Table 3:** Donor / recipient conditions and their relationship to the cortical capillary blood flow value. Multivariate analysis.

Type III test of Fixed Effects				
Source	Numerator df	Denominator df	F	Sig.
Intercept	1	91.4	53.22	.000
Donor^&^	1	106.1	17.56	.000
DGF^#^	1	109.9	0.37	.544
Donor´s hypertension	1	107	0.33	.569
Acute rejection	1	84.9	3.19	.078
Donor´s age	1	109.4	12.38	.001
Creatinine*	1	184.8	4.90	.028

^&^: living vs deceased donor.

^#^: Delayed graft function.

*: Serun creatinine and ultrasonography performed simultaneusly.

### Ability of CCBF Relative to Simultaneous Serum Creatinine for Improving Renal Function Prediction after 3 Years

CCBF levels measured at ROIs 2 and 3 in the proximal cortex and ROI 4 in the deep cortex were averaged to obtain the mean graft CCBF and correlate this value with that of plasma creatinine values and glomerular filtration rates (GFR) in the medium and long term (3^rd^ year).

CCBF values at months 1, 3 and 12 were significantly correlated. There was a significant correlation between averaged CCBF levels measured at month 1 and plasma creatinine levels (r, -0.49), GFR measured by 24-hour urine creatinine clearance (r, 0.43) and estimated GFR measured by MDRD (r, 0.49) at month 3 (p<0.05). However, there was no correlation with these parameters measured later on during the follow-up.

### Early-Stage Kidney Function Predictive Capacity

**Fig 8:**
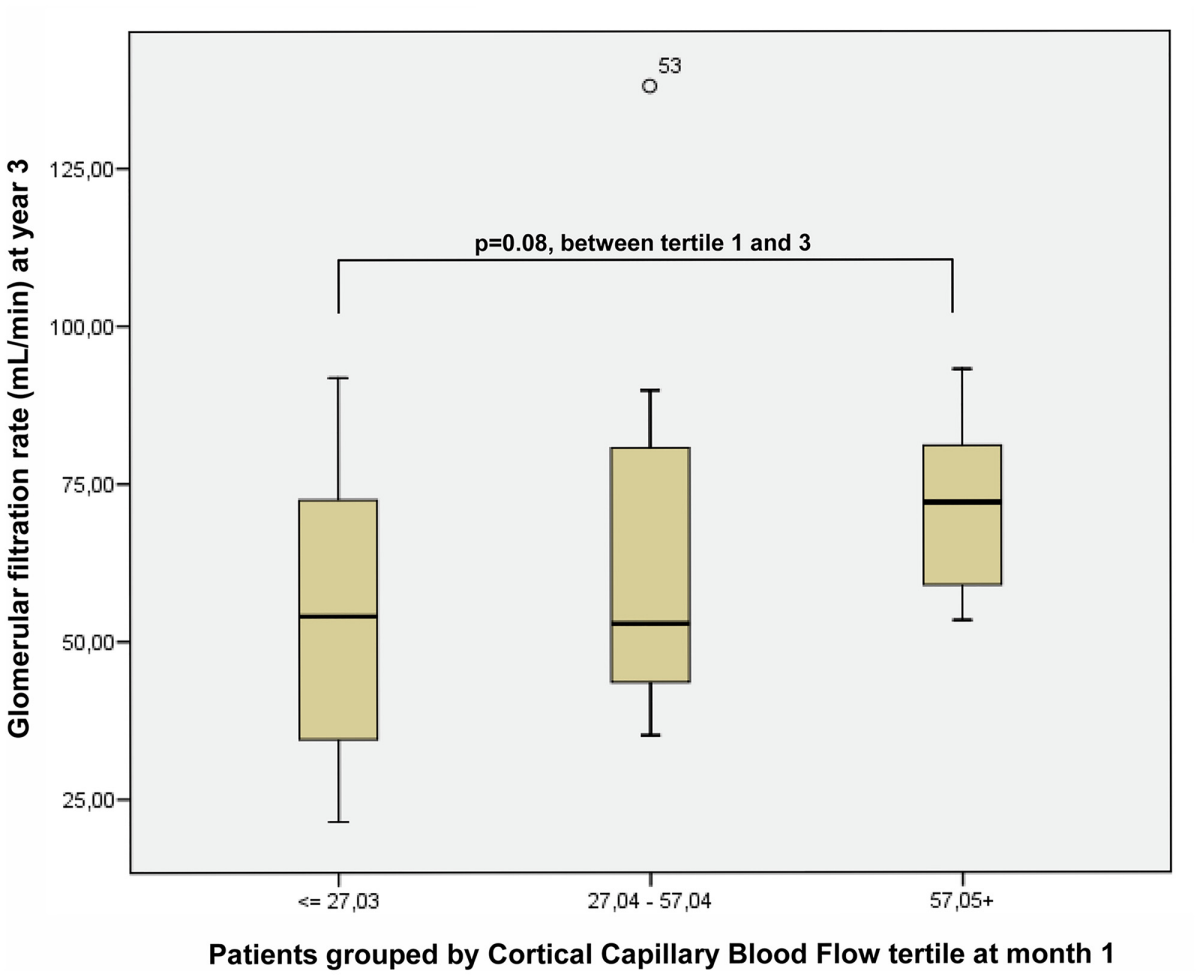
Box diagrams representing GFR values reached at year 3 by various groups of patients, grouped by CCBF tertile at month 1.

Plasma creatinine levels at month 1 were significantly correlated with plasma creatinine levels and GFR at all measured points until year 3. However, when patients with CCBF in the highest tertile (>44 dB/sec) at month 1 were compared with those in the lowest tertile (<25 dB/sec), both groups had similar creatinine levels (1.8–1.9 mg/dL). The first group achieved higher renal function in all measurements from month 3 to year 3 (creatinine mean value, 1.4 vs. 1.7 mg/dL; GFR, 52 vs. 68 mL/min; p<00000, for recipients with plasma creatinine levels <1.8 mg/dL).Fig 8 shows the box diagrams representing the GFR values reached at year 3 by the various patient groups, grouped by CCBF tertile at month 1.

### Late-Stage Kidney Function Predictive Capacity

**Fig 9:**
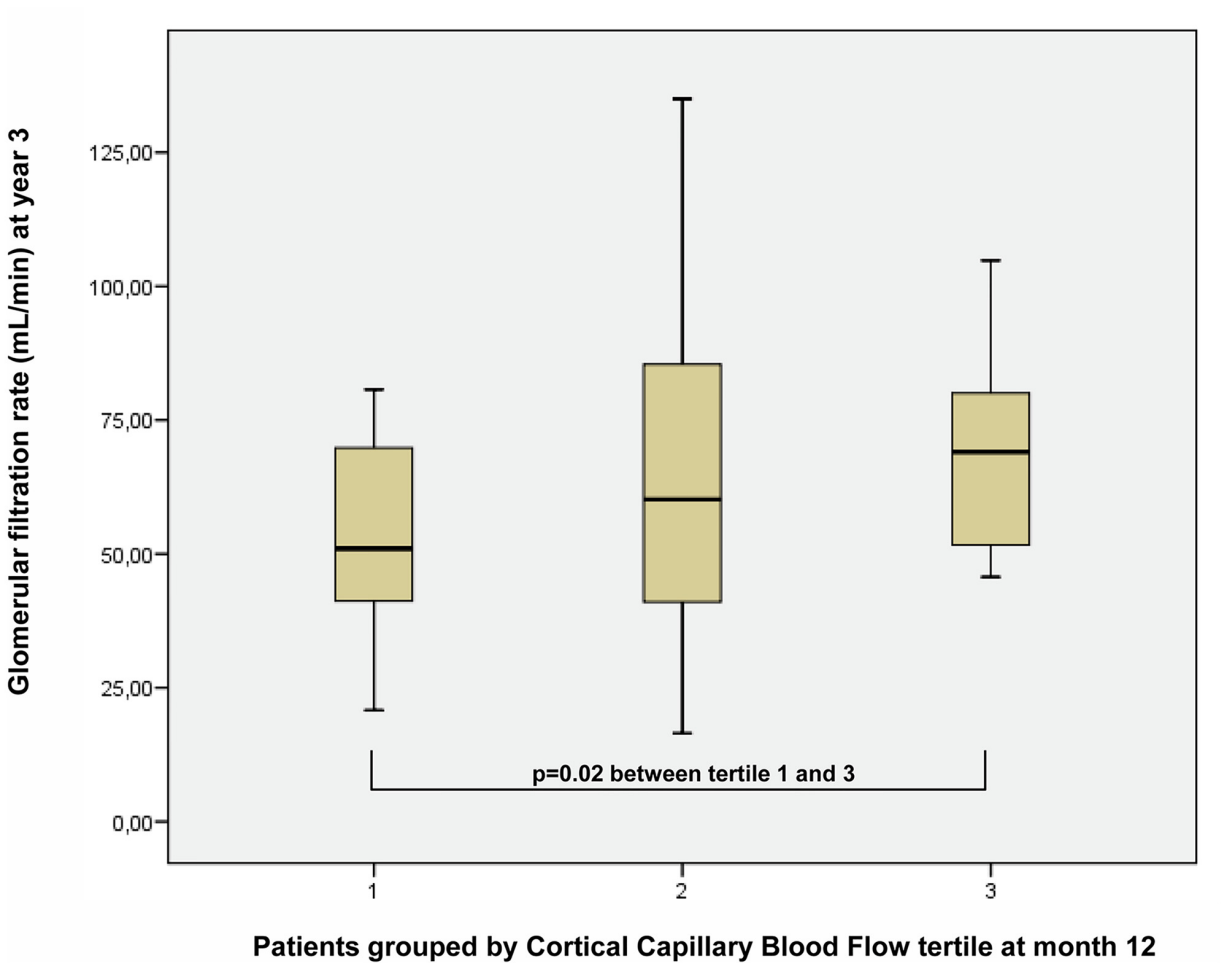
Patients with CCBF on the highest tertile compared with those in the lowest tertile at year 1, with similar plasma creatinine levels. The first group reached a significant higher GFR at year 3 (mean GFR value, 70 vs. 48 mL/min).

Plasma creatinine levels at year 1 were significantly correlated with plasma creatinine levels and GFR at all measured points until year 3. There was also a significant positive correlation between CCBF at year 1 and GFR at years 1 and 3. Once again, when patients with CCBF in the highest tertile (>38 dB/sec) were compared with those in the lowest tertile (<26 dB/sec) at year 1, there was no difference in mean plasma creatinine levels (1.3 and 1.5 mg/dL, respectively). The first group achieved a significant higher GFR at year 3 (mean GFR, 70 vs. 48 mL/min; p<00001; for recipients with plasma creatinine levels <1.8 mg/dL) (Fig 9).

CCBF had consistently better predictive ability than plasma creatinine. CCBF values correlate throughout the study period, whereas long-term plasma creatinine levels correlate well with month 1 plasma creatinine levels but not with intermediate levels.

## Discussion

This study demonstrates that an innocuous examination such as RT-CES is able to accurately quantify and iteratively measure cortical graft microcirculation in kidney recipients. Others authors have communicated in elegant studies their experience with graft RT-CES, even with histological correlation in transversal studies [19], standing out the interest of this procedure. This paper is the first to longitudinally analyze kidney recipients during their first year and could become a reference for clinicians who would like to use contrast-enhanced sonography in this field. This technique measures CCBF, which can be more effective than using plasma creatinine levels to predict long-term graft function. We therefore consider RT-CES a worthwhile complementary procedure for evaluating kidney recipients and that it occasionally outperforms other imaging methods by providing actual functional renal cortical values.

### The Natural History of CCBF

Our results suggest that CCBF has wide interpatient variability [19,21,28]and undergoes significant changes in the graft during the first year of follow-up. The first week is the most difficult period for interpreting CCBF results. However, from then on and especially after the first month, CCBF could reflect the graft’s actual vascular capacity and reserve [6,7]. The finding of the direct relationship between graft CCBF with donor age supports this conclusion CCBF values represent an added value for the examination of these uncertain situations (circulatory-death donors), which usually require renal biopsy. These donors, albeit younger, usually experience DGF over days or weeks thereby requiring iterative biopsies. It is notable that the long-term survival of grafts from these donors is higher than that of grafts from brain-death [29]. It is also worthy of note that the 4 recipients in our study with these conditions had normal CCBF values (similar to those of living donors) with no renal function at all. Serum creatinine levels are not a predictive marker for this situation. Living donors, given their expected minimal ischemia-reperfusion damage, can be considered a reference for normality. We believe that better CCBF in circulatory-death donors compared to brain-death donors is consequence of that the first ones are younger than 45 years who suffered a cardiac arrest in the street [29], and consequently have better peripheral vascular tree than those brain-death donors and circulatory-death donors taken at the hospital after a limitation of therapeutic resources. Data from circulatory-death donor grafts are purely indicative and descriptive because represent only four patients. However, although descriptive, we want to stand out our experience with these four patients because from clinical point of view, RT-CES visualization in these patients was unexpected and it was a surprise for us.

We should recognize the significant interpatient variability [19,21,28], which requires that this procedure be performed with the patient at their own control.

### The Effects of the Recipient on CCBF

The absence of anticalcineurin drugs was associated with a higher initial CCBF^20^. However, the later use of these drugs had similar results to those of patients who were not treated with these drugs. The vasoconstrictor effect appears to be transitory, and a negative effect from tacrolimus should be considered in the recovery phase of tubular necrosis and delayed graft function.

Acute rejection is associated with lower CCBF in general, as a result of tissue edema and infiltration. However, CCBF did not demonstrate sufficient sensitivity to distinguish rejection [18], although our study was not designed to specifically determine these differences.

Naesens M. et Al [30] established that graft resistive index measured by doppler ultrasound reflects more the characteristics of the recipient and less that of the graft. They observed in their prospective study that “*there was no correlation between the estimated GFR and the RI at 3 or 24 months*. *At 12 months there was a weak correlation (r*:*0*,*17*, *p*:*0*,*01)*”. When they tried to associate RI with histologic findings, in protocol biopsies, “*there was no significant or consistent correlation”*. Beside, RI was associated with patient survival but not with graft survival. We have not found correlation between resistive index and CCBF at any time (data not shown), possibly because CCBF reflects both donor and graft characteristics as it can be deduced by the CCBF correlation with donor´s age, acute rejection episodes, and living *vs*. brain-death donor and not with recipient individuality. We speculate that CCBF could reflect first the basal vasculature state from the donor and that afterwards this state changes by several inflammatory insults after ingraft.

### CCBF, when Compared with Simultaneous Serum Creatinine Levels, Improved Renal Function Prediction after 3 Years

The medium-term (3 years) prediction of graft function is of interest because it determines the whole graft project. Nonextreme serum creatinine levels at various points during the graft function follow-up have limited prognostic value. However, we have been able to demonstrate CCBF’s greater predictive value at month 1 and year 1 and for GFR at year 3 relative to serum creatinine levels, specifically for plasma creatinine values lower than 2 mg/dL. Serum creatinine levels are determined by GFR at any time. Its ability to predict overall graft outcome is intrinsically limited by the circumstances that condition GFR. However, it is recognized that the level achieved by the first year has a certain predictive capacity for medium-term graft outcome. Previous creatinine levels do not have this ability, except when GFR is low or zero, in which case the creatinine levels remain high, signaling a lack of renal function.

In contrast, both isolated and grouped CCBF values over the first year showed a peculiar behavior with predictive capacity. It remains to be seen whether these results can be extended to the future, allowing us to predict long-term renal function using CCBF, thereby converting it into an innocuous marker of great importance.

Since this is a pilot study, RT-CES cannot surpass conventional methods for assessing graft function such as serum creatinine measurement or proteinuria. By the present RT-CES is a useful, inoffensive and iterative tool to explore graft wellness and induce suspicion that indicates other invasive diagnostic methods.

### Limitations

Our study had a limited number of patients at a single center. Due to the significant interpatient variability, a larger study population would be desirable. The study was performed under standard clinical practice, although it was not controlled or randomized. So, we recognize in Table 2 that RT-CES was not performed in every patient at every time. However, more than 40 measures were performed at each point. Accordingly, appropriate statistical methods such as mixed regression models have been applied for this longitudinal study for these conditions. None data have been excluded what permits us to express all results with honesty. Coadministred drugs (hypotensor, immunosuppresor and other usual drugs) and their frequent doses changes did not take into account because the usual clinical modification in the more than 20 consultations in every patient during the first year after transplantation. These changes can´t be analyze in the limited number of patients. The technique used is not universally standardized, and there is a lack of studies to compare these results. We used a continuous contrast perfusion, whereas other authors have used bolus for contrast administration. In our study, the predictive value of CCBF was limited to the third year after transplantation; it would be desirable to establish its value over longer periods (e.g., 10 years).

## Conclusion

RT-CES is a tool that can accurately quantify and iteratively measure cortical graft microcirculation in kidney recipients. We have described the natural history of cortical capillary blood flow under regular clinical conditions and shown how it could become a reference for clinicians who use contrast-enhanced sonography in this field. The novelty of this study lies in its longitudinal nature in contrast with other cross-sectional studies published until now. The CCBF data are consistent with the clinical variables evaluatedand therefore in line with current knowledge. More studies with a homogeneous design are warranted to confirm these results.

## Supporting Information

S1 Video Real-time contrast-enhanced sonography from a recipient during the first year. Each videoclip has been performed at a different time from the ingraft (48 hours, day 7, month 1, month 3, month 12). The patient received a kidney from a 40-year-old male brain-death donor. The recipient was a 33-year-old man, and this was his second graft. He did not experience delayed graft function or acute rejection.
(MP4)
